# Inflammation in HIV-Infected Patients: Impact of HIV, Lifestyle, Body Composition, and Demography – A Cross Sectional Cohort Study

**DOI:** 10.1371/journal.pone.0051698

**Published:** 2012-12-10

**Authors:** Anne Langkilde, Janne Petersen, Henrik Hedegaard Klausen, Jens Henrik Henriksen, Jesper Eugen-Olsen, Ove Andersen

**Affiliations:** 1 Clinical Research Centre, Copenhagen University Hospital, Hvidovre, Denmark; 2 Department of Clinical Physiology and Nuclear Medicine, Copenhagen University Hospital, Hvidovre, Denmark; 3 Department of Infectious Diseases, Copenhagen University Hospital, Hvidovre, Denmark; South Texas Veterans Health Care System and University Health Science Center San Antonio, United States of America

## Abstract

**Objectives:**

To examine mechanisms underlying the increased inflammatory state of HIV-infected patients, by investigating the association of HIV-related factors, demography, lifestyle, and body composition with the inflammatory marker soluble urokinase plasminogen activator receptor (suPAR).

**Methods:**

suPAR was measured in EDTA-plasma and associated with HIV-related factors (HIV-duration, combination antiretroviral treatment (cART), nadir CD4+ cell count, CD4+ cell count, and HIV RNA); demography; lifestyle; and body composition determined by Dual energy X-ray Absorptiometry (DXA) scan, in multiple linear regression analyses adjusted for biological relevant covariates, in a cross-sectional study of 1142 HIV-infected patients.

**Results:**

Increased suPAR levels were significantly associated with age, female sex, daily smoking, metabolic syndrome and waist circumference. cART was associated with 17% lower suPAR levels. In cART-treated patients 10-fold higher HIV RNA was associated with 21% higher suPAR, whereas there was no association in untreated patients. Patients with CD4+ cell count<350 cells/µL had 7% higher suPAR, but we found no association with nadir CD4+ cell count or with duration of HIV-infection. Finally, suPAR was not associated with adipose tissue distribution, but strongly associated with low muscle mass. In patients infected through intravenous drug use (IDU), CD4+ cell counts<350 cells/µL were associated with 27% lower suPAR (p = 0.03), and suPAR was 4% lower pr. year during treatment (p = 0.05); however, there was no association with HIV RNA, duration of HIV-infection, nor cART.

**Conclusion:**

We found elevated suPAR levels in untreated patients compared to patients on cART. Moreover, we observed a significant positive association between suPAR and HIV RNA levels in cART-treated patients. Age, HIV-transmission through IDU, metabolic syndrome, smoking, and low leg muscle mass were also significantly associated with suPAR levels. Our study therefore indicates, that also other aspects of living with HIV than virologic and immunologic markers add to the increased inflammation in HIV-infected patients.

## Introduction

Introduction of combination antiretroviral therapy (cART) has dramatically increased the lifespan of HIV-infected patients and decreased the prevalence of AIDS-related deaths [1]. The lifespan is still lower than that expected for non-HIV-infected individuals [2]–[4], because of both AIDS-related and non-AIDS related mortality. Several studies have reported higher prevalence of comorbidities such as cardiovascular disease (CVD), type 2 diabetes, and possibly cancer than in the general population [5]–[7]. What underlies the increased prevalence of non-AIDS-related comorbidities is not fully understood; but coinfections such as Hepatitis C [4], cART [8]; late HIV-diagnosis [3], [4]; lifestyle [9], [10], and inflammation [11] have all been implicated.

Inflammation affects metabolism [12] and increased inflammation has been demonstrated to be a risk factor for CVD, type 2 diabetes, cancer and overall mortality in the general population [13]. HIV-infection causes chronic immune activation, and HIV-infected patients are characterised by higher inflammatory levels than non-HIV-infected individuals [14]. It has been proposed, that accelerated ageing characterises long term HIV-infection [15].

Urokinase plasminogen activator receptor (uPAR) is a membrane bound receptor involved in numerous processes such as fibrinolysis, cell migration and cell signalling [16]. uPAR is expressed on activated T cells and macrophages among other cells, and its expression is upregulated by HIV [17]. Increased levels of the soluble form of uPAR (suPAR) have been found in various infectious, inflammatory, autoimmune and malignant diseases, and suPAR levels generally associate with disease severity [17]–[20]. suPAR levels correlate positively with tumor necrosis factor alpha (TNF-α), leukocyte numbers, and C-reactive protein (CRP) [21], [22], and suPAR seems to be a stable marker of inflammation, with low diurnal variation and stable *in vitro* properties [21], [23]. We have previously shown that suPAR was associated with dysmetabolism in HIV-infected patients and mortality in the pre-cART era [21], [24]. Moreover, increased suPAR levels were associated with type 2 diabetes, CVD, cancer, and mortality in a prospective general population-based study [22], [25].

In this study, we investigated whether HIV-related factors, demography, lifestyle and body composition were associated with inflammation measured by suPAR, thereby exploring mechanisms underlying the decreased life-time expectancy of HIV-infected patients. We found suPAR to associate with established risk factors for cardiovascular disease and non-AIDS-related mortality, and to reflect other aspects of HIV-disease than immunologic and virologic markers.

## Methods

### Ethics Statement

All patients included gave written informed consent to have an extra blood sample collected during routine HIV-management to be used for future HIV research. The Danish Data Protection Agency approved the storage and collection of samples (protocol: 2007-41-1634), according to Danish law, only the Danish Data Protection Agency needs to approve this. The local Ethics committee for the Capital Region of Denmark and the Danish Data Protection Agency approved the use of: stored blood samples; data from patients’ medical records; Dual energy X-ray absorptiometry (DXA) scans; routine blood tests; and patient administered questionnaire for this study (protocols: H-4-2012-008 and 2007-58-0015, respectively). The study was performed according to the Declaration of Helsinki.

### Setting

In 2007, approximately 3780 (0.07% of the overall population) adults were living with HIV in Denmark [26]. Medical care and cART is tax-paid and free of charge, and provided at few specialised centres. Approximately 37% of HIV-infected patients in Denmark attended the Department of Infectious Diseases, Copenhagen University Hospital, Hvidovre for disease management in 2007 [26].

The criteria for initiating cART in 2007 were: acute HIV-infection, pregnancy, CD4+ cell counts<300 cells/µL, HIV-related disease, and until 2001 also: plasma HIV RNA>100 000 copies/mL. A routine annual metabolic monitoring program was introduced at Department of Infectious Diseases, Copenhagen University Hospital, Hvidovre, in 2004, where patients were enrolled on a first come, first served basis. The metabolic monitoring programme contains blood measurements, DXA scans and standardised patient questionnaire.

### Population

Patients were recruited from the out-patients clinic at Department of Infectious Diseases, Copenhagen University Hospital, Hvidovre, if they were ≥18 years, and were seen at the out-patient clinic during 2007.

### Data Collection

Data were obtained from patients’ medical records. Blood tests were performed as part of the routine HIV-management and annual metabolic monitoring. The lower level of detection for HIV RNA was 39 copies/mL. DXA scans and standardised patient questionnaire were performed as part of the metabolic monitoring programme. Blood tests for metabolic monitoring were obtained fasting and include glucose, lactate, lipid and cholesterol levels. The standard patient questionnaire includes information on predisposition to cardiovascular diseases and diabetes; anthropometry; tobacco use; blood pressure and patient reported lipodystrophy; described in [26].

**Figure 1:**
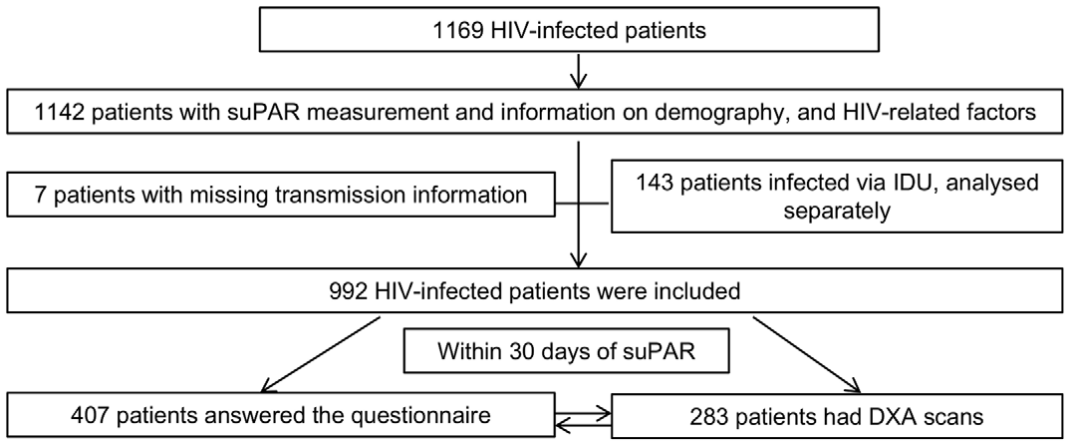
Flow chart of study cohort. 235 patients had both been DXA-scanned and answered the questionnaire. **Abbreviations:** DXA: Dual Energy X-ray Absorptiometry; IDU: Intravenous drug use; suPAR: soluble urokinase plasminogen activator receptor.

About 30% of the patients attended the metabolic monitoring programme, but not necessarily all parts of it, 235 patients were DXA scanned and answered the questionnaire. DXA scans and standardised patient questionnaires were not always performed, and not necessarily the same day as the blood test. We included data from DXA scans and standardised patient questionnaire if performed within 30 days of blood sampling, see Figure 1.

Nadir CD4+ cell count was defined as the lowest CD4+ cell count ever measured in the patient.

### Metabolic Syndrome

Metabolic syndrome was determined according to the updated National Cholesterol Education Programme (NCEP) Adult Treatment Panel (ATP) III, 2004 [27], if sufficient information was available. Three or more of the following five components should be present to diagnose metabolic syndrome: waist circumference >102 cm for men, >88 cm for women; fasting triglycerides ≥150 mg/dl; fasting HDL cholesterol: <40 mg/dl in men, <50 mg/dl in women; blood pressure: ≥130/≥85 mmHg; fasting plasma glucose ≥5.6 mM.

### Body Composition evaluated by DXA scans

Patients were DXA-scanned with a Norland XR-36 (Gammatec A/S, Værløse, Denmark). Lipoatrophy was assessed by evaluating the peripheral fat per cent defined as the fat mass of arms and legs divided by the total mass of arms and legs. Lipodystrophy was assessed by evaluating the ratio of trunk fat per cent (trunk fat mass/total trunk mass ×100) to leg fat per cent (fat mass of legs/total mass of legs ×100) [28].

### suPAR Measurements

EDTA-blood samples were taken at routine visits at the Outpatients clinic; plasma was separated and stored at −20°C. suPAR was measured in duplicates using the suPARnostic™ ELISA (ViroGates®, Birkerød, Denmark). The suPARnostic^TM^ ELISA has been validated to measure concentrations of 0.6–22 ng/mL. The inter-assay variance of a control sample was 20%, and the intra-assay variance of duplicate measurements of samples was 3.7%. Samples were measured again, if the coefficient of variation>10%.

### Statistics

We investigated the association of suPAR with demography, HIV-related factors, lifestyle, and body composition using univariate and multiple linear regression. Multiple regression analyses were adjusted for biological relevant covariates. All multiple analyses were adjusted for age, sex and descent (European vs. non-European). Smoking was assessed as daily smoking vs. non-daily smoking. Patients without HIV-transmission information were not included in analyses. Patients infected through intravenous drug use (IDU) were analysed separately, since this group of patients differs according to lifestyle, comorbidities, treatment compliance and treatment initiation [29]–[31].

We tested Goodness of fit of models for normal distribution of residuals and homogeneity of variances. suPAR was transformed using log_2_(x) to obtain normally distributed residuals, results are back-transformed using 2^x^, and therefore showed as % estimates. Viral load (VL) was transformed using log_10_(x) due to its wide range.

The Statistics programme “Statistical Analysis Systems” (SAS, version 9.2; SAS Institute, Cary, NC, USA) was applied for analyses. We considered P values less than 0.05 for statistical significant.

## Results

### Cohort Description

We included 1142 HIV-infected patients in this study, see Figure 1. The associations for patients reporting to be infected through intravenous drug use (N = 143) were analysed separately and are reported in the paragraph “Patients Infected through IDU”.

Baseline characteristics of patients are seen in Table 1. Men comprised 75% of the cohort, and 76% were of European descent. The median duration of HIV-infection was 9 years. Eighty-five per cent received antiretroviral treatment, and 99.9% of these were treated with a combination of three or more antiviral drugs, the median treatment duration was 7 years. Thirty-five per cent of patients smoked daily.

**Table 1:** Baseline characteristics for HIV-infected patients not infected through intravenous drug use (IDU).

Demography	Median	Range (5%; 95% percentiles)	N total
Age (years)	44.3	29.5; 64.2	992
Sex (men)	74.9%		992
European descent	76.0%		992
**HIV-related Factors**			
HIV duration (years)	9.2	0.6; 21.8	992
Nadir CD4 (cells/µL)	183.0	9.0; 476.0	959
Nadir CD4<200 cells/µL	55.6%		959
Current cART	84.7%		990
Never cART	13.0%		990
Total treatment duration (years)	6.7	0.5; 10.8	861
CD4<350 cells/µL	21.2%		949
HIV RNA (copies/mL)	39.0	39.0; 5110.0	942
HIV RNA≤40 copies mL	72.8%		942
**Lifestyle**			
Current daily smoking	35.1%		428
Waist circumference (cm)	91.5	72.0; 110.5	415
BMI (kg/m^2^)	23.9	19.1; 31.7	465
Metabolic syndrome	30.6%		366

**Abbreviations:** cART: Combination antiretroviral treatment; BMI: Body mass index.

### suPAR and Demography

Results from univariate and multiple regression analyses are seen in Table 2. suPAR levels were 7% lower in men than women (p = 0.02), when adjusted for age and European descent. Higher age was significantly associated with higher suPAR levels in both uni- (p<0.001) and multiple regression analyses (p<0.001). Patients of European descent had 10% higher suPAR levels than patients of other descent (p<0.001), also when adjusted for sex and age (p = 0.004). More patients of European descent than patients of other descent (38.3% vs. 19.2%), and more men than women (38.9% vs. 19.8%) smoked daily. When further adjusting the analyses for daily smoking in the 428 patients with information on smoking, the estimate for European descent changed from 9% (p = 0.11) to 5% (p = 0.30), and the estimate for men vs. women changed from −7% (p = 0.12) to −10% (p = 0.02).

**Table 2:** HIV- and non HIV-related factors influencing suPAR levels.

	Univariate			Multiple		
Variables	% Estimate (95% CI)	P	N	% Estimate (95% CI)	P	N
**Demography**						
Age ≥60 vs. <40 years	20.0 (10.6; 30.2)	<0.001	992	19.1 (9.4; 30.0)	<0.001	992
Age ≥60 vs. 40–50 years	9.3 (1.0; 18.3)			8.4 (0.1; 17.4 )		
Age ≥60 vs. 50–60 years	5.2 (−3.6; 14.8)			4.7 (−4.0; 14.3)		
Sex (men vs. women)	−1.8 (−6.9; 3.7)	0.52	992	−7.1 (−12.8; −1.1)	0.02	992
European descent	10.1 (4.4; 16.3)	<0.001	992	9.8 (3.1; 17.0)	0.004	992
**HIV-related**						
HIV duration (years)	0.5 (0.1; 0.8)	0.01	992	0.1 (−0.3; 0.5)	0.75	992
Nadir CD4+ (cells/µL)*	0.01 (−0.01; 0.02)	0.44	959	−0.01 (−0.02; 0.01)	0.62	958
No current cART*	9.0 (2.2; 16.2)	0.009	990	17.3 (8.0;27.4)	<0.001	958
Treatment duration (years)**	0.3 (−0.4; 1.0)	0.43	861	−1.4 (−2.3; −0.4)	0.006	845
CD4<350 vs. 350≥cells/µL***	9.1 (2.8; 15.6)	0.004	949	6.6 (−0.1; 13.8)	0.05	907
VL, cART-treated patients (pr. 10-fold)***	18.9 (11.8; 26.5)	<0.001	794	20.5 (13.1; 28.4)	<0.001	779
VL, for untreated patients (pr. 10-fold)***	3.7 (−6.0; 14.5)	0.46	146	2.4 (−8.7; 14.9)	0.68	128
**Lifestyle**						
Daily vs. no daily smoking	26.4 (18.3; 35.1)	<0.001	428	26.7 (18.6; 35.4)	<0.001	428
Waist circumference (cm)#	0.2 (−0.04; 0.5)	0.09	415	0.3 (0.02; 0.6)	0.03	408
BMI <20 vs. 20–25 (kg/m^2^)##	2.9 (13.2; 6.5)	0.01	483	−0.8 (−8.8; 11.4)	0.07	408
BMI <20 vs. 25–30 (kg/m^2^)##	11.9 (1.0; 23.9)			7.5 (−5.3; 22.0)		
BMI <20 vs. ≥30 (kg/m^2^)##	−5.8 (−17.6; 7.9)			−6.6 (−22.4; 12.4)		
Metabolic syndrome#	6.6 (−1,8; 15.8)	0.13	366	8.4 (0.2; 17.2)	0.04	338

All multiple analyses are adjusted for sex, age, and European descent.

* Multiple analyses are also adjusted for time since HIV-diagnosis.

** Multiple analyses are also adjusted for time since HIV-diagnosis, current treatment and nadir CD4+ cell counts.

*** Multiple analyses are also adjusted for time since HIV-diagnosis, nadir CD4+ cell counts, current treatment, CD4+<350 vs. ≥350 cells/µL, and log_10_(VL).

# Multiple analyses are also adjusted for daily smoking.

## Multiple analyses are also adjusted for daily smoking, waist circumference.

**Abbreviations:** cART: Combination antiretroviral treatment; VL: viral load; BMI: Body mass index; CI: Confidence interval.

### Impact of HIV-related Factors on suPAR levels

We explored the association of components of HIV-disease with suPAR levels, to examine HIV-disease-specific factors influencing inflammation. Time since HIV-diagnosis was significantly associated with suPAR levels in univariate analysis (estimate = 0.5%, p = 0.01), but not when adjusted for sex, age and European descent (estimate = 0.1%, p = 0.75). There was no association of nadir CD4+ cell count and suPAR levels. Untreated patients had 17% higher suPAR levels than those receiving cART in multiple regression analyses, see Table 2 (p<0.001).

We assessed the association between treatment duration and suPAR levels in patients on current cART. suPAR levels decreased with total treatment duration (estimate = −1% pr. year, p = 0.006), when adjusted for sex, age, European descent, duration of HIV-infection, and nadir CD4+ cell counts. Patients with CD4+ cell counts<350 cells/µL had 7% higher suPAR levels (p = 0.05) than patients with higher CD4+ cell counts in multiple analyses.

**Figure 2:**
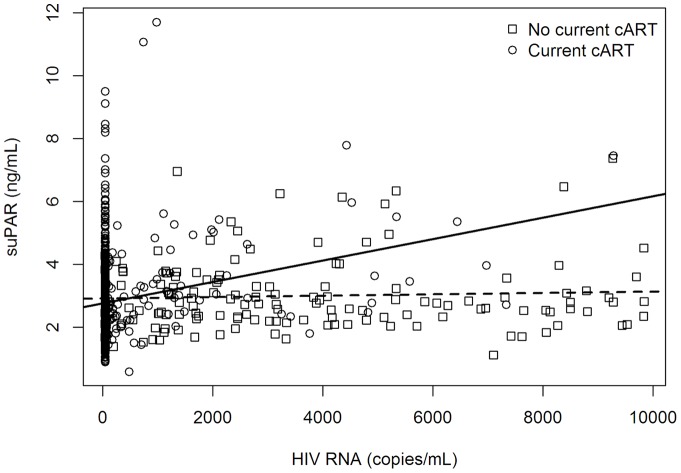
The association of suPAR and viral load according to treatment status. The figure represents a scatter plot of the association between suPAR and viral load. Circles represent cART-treated patients (N = 838); boxes represent non cART-treated patients (N = 152). The regression line for cART-treated patients is continuous; the regression line for non-cART treated patients is dashed. The lower level of detection of HIV RNA in this study was 39 copies/mL. **Abbreviations:** cART: Combination antiretroviral treatment; suPAR: soluble urokinase plasminogen activator receptor.

A 10-fold higher VL was associated with 21% higher suPAR levels (p<0.001) in cART-treated patients, but not in untreated patients (estimate = 2%, p = 0.68), when adjusted for sex, age, European descent, duration of HIV-infection, nadir CD4+ cell counts, and CD4+ cell counts, see Figure 2 and Table 2.

### Influence of Lifestyle on suPAR levels

Patients smoking daily had 27% higher suPAR levels (p<0.001) than patients not smoking daily in multiple analysis adjusted for sex, age, and European descent. Large waist circumference was significantly associated with high suPAR levels (estimate = 3%, p = 0.03 pr. 10 cm) adjusted for sex, age, European descent, and daily smoking. BMI was significantly associated with suPAR levels in univariate analyses (p = 0.01) but not when adjusting for sex, age, European descent, waist circumference and daily smoking. Metabolic syndrome could be assessed in 366 patients, and we found that patients with metabolic syndrome had 8% higher suPAR levels (p = 0.04) when adjusting for sex, age, European descent, and daily smoking.

### The Influence of DXA-measured Body Composition on suPAR levels

We analysed the association of suPAR with measures of fat mass and lean mass in the subgroup of 283 patients with DXA scans, see Table 3. High suPAR levels were significantly associated with low total lean mass/height (kg/m^2^) (p = 0.02 in multiple analysis). When assessing the leg lean mass specifically, we found a more pronounced association (estimate = −9%, p<0.001 pr. kg/m^2^) in multiple analyses. There was no significant association between suPAR levels and total fat per cent, total fat mass or regional fat mass distribution, neither in univariate or multiple analyses adjusted for sex, age, European descent, and daily smoking.

**Table 3:** Subgroup analyses of body composition and suPAR levels in patients with DXA scan.

	Univariate			Multivariate		
Body Composition	% Estimate (95% CI)	P	N	% Estimate (95% CI)	P	N
Lean mass/h^2^ (kg/m^2^)	−1.8 (−3.4; −0.1)	0.04	283	−2.3 (−4.2; −0.4)	0.02	283
Lean mass_leg_/h^2^ (kg/m^2^)	−9.0 (−13.0; −4.9)	<0.001	283	−9.1 (−13.3; −4.8)	<0.001	283
Fat mass/h^2^ (kg/m^2^)	0.1 (−1.4; 1.6)	0.93	283	1.3 (−0.6; 3.3)	0.17	234
Total fat%	0.1 (−0.4; 0.5)	0.76	283	0.4 (−0.2; 1.1)	0.19	234
Limb fat%	0.2 (−0.7; 1.0)	0.69	283	0.8 (−0.4; 2.1)	0.20	234
Trunk fat %/leg fat %	−7 (−22; 10)	0.39	283	−8.8 (−23.8; 10.7)	0.37	234

Multiple analyses of lean mass measures are adjusted for sex, age and European descent. Multiple analyses of fat mass measures are adjusted for sex, age, European descent, and daily smoking.

**Abbreviations:** CI: Confidence interval; h = height.

### Patients Infected through IDU

Men comprised 55% of this group and 93% were of European descent. The median age was 43 years (5 percentile: 31 years; 95 percentile: 55 years), and the median duration of HIV-infection was 11 years (5 percentile: 11 months; 95 percentile: 22 years). Seventy-five per cent received cART and the median duration was 5 years (5 percentile: 4 months; 95 percentile: 10 years); 93% of cART-treated patients had VL<400 copies/mL, and 79% had VL≤40 copies/mL. Eleven per cent of patients had CD4+ cell count<200 cells/µL; 33% had CD4+ cell count<350 cells/µL. Nadir CD4+ cell count was<200 cells/µL in 59% of the patients.

Patients infected through IDU had significantly higher suPAR levels (median suPAR level: 4.7 ng/mL, range 21.1 ng/mL), than patients reporting to be infected through other routes (median suPAR level: 2.6 ng/mL, range 11.1 ng/mL, p<0.001), also when adjusted for sex, age and European descent (estimate = 62%, p<0.001).

suPAR levels were significantly associated with higher age in multiple analyses (p = 0.02); but there was no significant association with sex or ethnicity (p = 0.63 and p = 0.26, respectively). There was no significant association of duration of HIV-infection and suPAR levels in multiple analysis adjusted for sex, age and European descent (estimate = −0.1%, p = 0.92). cART status did not affect suPAR levels (estimate = 7%, p = 0.74 for not receiving cART), nor did nadir CD4+ cell counts (estimate = −0.005%, p = 0.92 pr. cell/µL), adjusted for sex, age, European descent, HIV duration, nadir CD4+ cell counts and treatment, respectively. Long treatment duration was negatively associated with suPAR levels in multiple analyses adjusted for sex, age, European descent, HIV duration, and nadir CD4+ cell counts (estimate = −4.2%, p = 0.05, 95%CI: −8.2%; −0.1% pr. year). The association with CD4+ cell count and VL was assessed in multiple analyses adjusted for sex, age, European descent, HIV duration, nadir CD4+ cell counts, treatment, log_10_(VL) and CD4+ cell counts, respectively. Patients with CD4+ cell counts≥350 cells/µL had 27% lower suPAR levels (p = 0.03; 95% CI: −44%, −3%) than patients with CD4+ cell counts<350 cells/µL, and there was no significant association with VL (estimate = 10% pr. 10-fold increase, p = 0.42).

## Discussion

We identified HIV-related and non-HIV-related factors influencing suPAR levels, adding to the growing knowledge of inflammation and HIV-disease in the cART era. Increased suPAR levels were significantly associated with higher age, female sex, metabolic syndrome, daily smoking, low leg muscle mass, and higher waist circumference; but not significantly associated with BMI. These findings are in accordance with previous findings in the general population [22], [32]. European descent was associated with higher suPAR levels in multiple analyses; however not when performing subgroup analyses adjusted for daily smoking. Thus, a higher daily smoking frequency among patients of European descent could explain the association of European descent and higher suPAR levels.

In addition to the inflammatory effects of demography, lifestyle and body composition, we assessed the association of HIV-related factors with suPAR. suPAR levels were 7% higher in patients with low CD4+ cell counts (<350 cells/µL), but we found no association of suPAR and duration of HIV-infection, nor with nadir CD4+ cell counts. Untreated patients had 17% higher suPAR levels than cART-treated patients (p<0.001), and low suPAR levels were weakly associated with longer treatment duration (estimate = −1%, p = 0.006 pr. year). This is in agreement with a previous follow-up study that found decreasing suPAR levels after cART-initiation during a 5-years period [33].

For every 10-Fold increase in VL, we found 21% higher suPAR levels in multiple regression analysis in cART-treated patients (p<0.001); however, there was no significant association in patients not receiving treatment (estimate = 2%, p = 0.68), see Figure 2. These findings could indicate that the dose-response relationship of suPAR and VL observed in cART-treated patients is not only due to VL-induced inflammation, but might be an effect of factors underlying high VL in cART-treated patients. Poor treatment-compliance could explain high VL in cART-treated patients. Psychosocial problems or substance abuse are more frequent in patients with low adherence [34], and the association we find might be an association of suPAR with comorbidities associated with psychosocial problems or substance abuse, resulting in poor cART-adherence and high VL. Supporting this, we found a 72% higher suPAR level, in patients reporting to be infected through IDU. However, VL still seems to affect suPAR levels, since we observe higher suPAR in untreated patients, and there could be a VL-threshold inducing systemic immune activation and inflammation reflected in increased suPAR levels.

suPAR was associated differently with HIV-related factors in patients infected through IDU. In this group of patients, we found 27% higher suPAR levels in patients with CD4+ cell counts<350 cells/µL, but no association with VL. If these patients are still active IDUs, other factors than VL could induce inflammation, such as frequent bacterial infections, and thereby decrease the importance of VL in inducing inflammation. Moreover, Hepatitis C (HCV) is more prevalent in this group of patients, and suPAR levels have been shown to increase with liver fibrosis in HCV-infected patients [35], [36]. Finally, HIV-infected patients infected through IDU have higher mortality risk [10], and initiate cART later, resulting in lower CD4+ cell gains [29].

We did not find any association with fat deposition measures by DXA scans. We have previously found increased suPAR levels in HIV-infected patients with clinician diagnosed lipodystrophy [37]. This divergence could reflect difficulties in assessing lipodystrophy using single DXA scans. Bonnet et al. [28] proposed reference values to define lipodystrophy by DXA scan. Only four of 283 patients in this study had lipodystrophy when applying this definition, indicating that it is not sensitive enough. Previous findings have found C-reactive protein to be more strongly associated with BMI and waist circumference than suPAR, whereas suPAR was found to be more strongly associated with poor outcome [22], [32]. Here, we did not find any significant association with BMI in multivariate analysis, but suPAR increased significantly with waist circumference (0.3% pr. cm) and patients with metabolic syndrome had 8% higher suPAR. Thus, suPAR levels are affected by central adiposity and metabolic syndrome.

For the first time, the association of suPAR and muscle mass was assessed. We found that suPAR was strongly associated with low leg muscle mass. We are not aware of any studies examining the role of suPAR and physical activity; however, numerous studies have demonstrated a strong link between exercise, muscles and inflammation [38]. We consider the association of suPAR and leg muscle mass to be an indirect association, in that decreased leg muscle mass has been shown to be an independent risk factor for 5-year mortality in HIV-infected patients [39], and increased suPAR levels also associate with mortality [22], [24]. However, we cannot exclude that the association of suPAR with low muscle mass is a physical activity-mediated effect.

The mechanistic role of suPAR in HIV-infection is still not fully understood due to the numerous functions and complex interplay between suPAR and uPAR, and their ligands: uPA, vitronectin, and integrins [16], [17]. Whether suPAR is merely a marker and/or directly causes disease still remains to be established. suPAR has been associated with a variety of diseases and disease progression [19], but to our knowledge suPAR has only been identified as a causal factor in one disease, focal segmental glomerulosclerosis [40]. In our opinion, suPAR does not reflect disease-specific pathology, but more likely, processes central to a variety of diseases. The results of this study and previous studies [17], [22], [24], [41] suggest that elevated suPAR levels in cART-treated and untreated patients have different causes. In untreated patients HIV-induced immune activation is likely to increase suPAR levels [17], [41]. In virally suppressed cART-treated patients, pathophysiologic processes associated with age; smoking; long term immune alterations caused by HIV; and low leg muscle mass, could be the prime suPAR inducers. Overall, suPAR could be a marker of accelerated ageing.

There are some limitations to this study. We did not assess the effect of co-infections and co-morbidity, since we did not have complete information. Co-infections have been shown to increase mortality rates [4], and co-infections could increase suPAR levels [19]. However, HCV co-infection is more prevalent in patients infected via IDU, and these patients were analysed separately.

If we had had complete information about lipid-lowering, anti-diabetic and blood pressure treatment, more patients could possibly have been diagnosed with metabolic syndrome. Furthermore, we assessed the association of suPAR and relatively high CD4+ cell counts (<350 cells/µL) in multiple regression analyses, and not <200 cells/µL, since only 54 patients infected by other routes than IDU had CD4+ cell counts<200 cells/µL. We expect that the association would be significantly stronger if we had evaluated CD4+ cell counts≥ vs. <200 cells/µL.

Our study adds to the growing knowledge of inflammation and HIV-disease in the cART era, by identifying factors that associate with inflammation. We found that increased suPAR levels were significantly associated with established risk factors for mortality and morbidity. Age, HIV-transmission through IDU, metabolic syndrome, daily smoking, and low leg muscle mass were associated with suPAR levels. However, we did not find any association with nadir CD4+ cell count, and suPAR was only 7% higher in individuals with low CD4+ cell count (<350 cells/µL). Moreover, high suPAR levels were associated with high VL only in cART-treated patients, indicating that factors causing or segregating with poor viral suppression in cART-treated HIV-infected patients could be associated with increased suPAR levels. Thus, it seems like suPAR reflects different aspects of living with HIV than CD4+ cell counts and VL, and that other aspects of living with HIV add to the increased inflammation in HIV-infected patients.
