# Correction: Inflammation in HIV-Infected Patients: Impact of HIV, Lifestyle, Body Composition, and Demography – A Cross Sectional Cohort Study

**DOI:** 10.1371/journal.pone.0094152

**Published:** 2014-04-09

**Authors:** The PLOS ONE Staff

The third and fourth sentences of the Results sub-section of the Abstract are incorrect. The correct sentences should read: In cART-treated patients 10-fold higher HIV RNA was associated with 15% higher suPAR, whereas there was no association in untreated patients. Patients with CD4+ cell count <350 cells/μL had higher suPAR levels than patients with CD4+ cell count ≥350 cells/μL, though not significantly. We found no association with nadir CD4+ cell count or with duration of HIV-infection.

The second to last sentence of the Results sub-section of the Abstract is incorrect. The correct sentence should read: Finally, suPAR was not associated with adipose tissue distribution, but strongly associated with low leg muscle mass.

The last sentence of the Results sub-section of the Abstract is incorrect. The correct sentence should read: In patients infected through intravenous drug use (IDU), CD4+ cell counts ≥350 cells/μL were associated with 27% lower suPAR (p  =  0.03), and suPAR was 4% lower pr. year during treatment (p  =  0.05); however, there was no association with HIV RNA, duration of HIV-infection, nor cART.

The last sentence of the second paragraph of the “Impact of HIV-related Factors on suPAR levels” sub-section of the Results is incorrect. The correct sentence should read: Patients with CD4+ cell counts <350 cells/μL had 6% higher suPAR levels (p = 0.09) than patients with higher CD4+ cell counts in multiple analyses.

The third paragraph of the “Impact of HIV-related Factors on suPAR levels” sub-section of the Results is incorrect. The correct sentence should read: A 10-fold higher VL was associated with 15% higher suPAR levels (p<0.001) in cART-treated patients, but not in untreated patients (estimate = 2%, p = 0.60), when adjusted for sex, age, European descent, duration of HIV-infection, nadir CD4+ cell counts, and CD4+ cell counts, see Figure 2 and Table 2.

**Figure 2:**
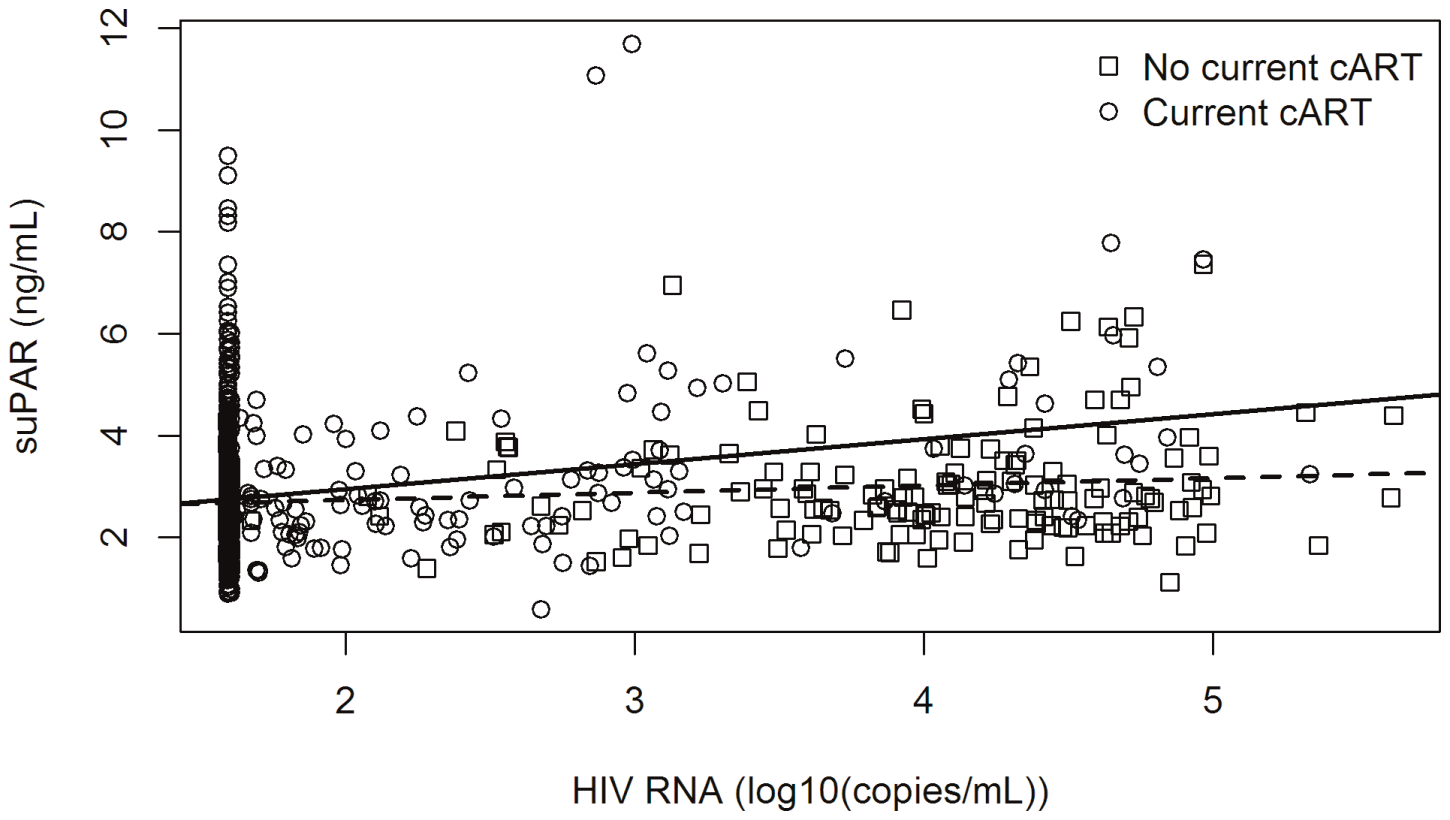
The association of suPAR and viral load according to treatment status. The figure represents a scatter plot of the association between suPAR and viral load. Circles represent cART-treated patients (N = 795); boxes represent non cART-treated patients (N = 150). The regression line for cART-treated patients is continuous; the regression line for non-cART treated patients is dashed. The lower level of detection of HIV RNA in this study was 39 copies/mL. **Abbreviations:** cART: Combination antiretroviral treatment; suPAR: soluble urokinase plasminogen activator receptor.

The last sentence of the “Patients Infected through IDU” sub-section of the Results is incorrect: The correct sentence should read: Patients with CD4+ cell counts ≥350 cells/μL had 27% lower suPAR levels (p = 0.03; 95% CI:-45%, -4%) than patients with CD4+ cell counts <350 cells/μL, and there was no significant association with VL (estimate = 5% per 10-fold increase, p = 0.57).

The second sentence of the second paragraph of the Discussion section is incorrect. The correct sentence should read: suPAR levels were 6% higher in patients with low CD4+ cell counts (<350 cells/μL), though not significantly. We found no association of suPAR and duration of HIV-infection, nor with nadir CD4+ cell counts.

The first sentence of the third paragraph of the Discussion section is incorrect. The correct sentence should read: For every 10-Fold increase in VL, we found 15% higher suPAR levels in multiple regression analysis in cART-treated patients (p<0.001); however, there was no significant association in patients not receiving treatment (estimate = 2%, p = 0.60), see Figure 2.

The first three sentences of the fifth paragraph of the Discussion section are incorrect. The sentences should read: We did not find any association with fat deposition measures or lipodystrophy by DXA scans. We have previously found increased suPAR levels in HIV-infected patients with clinician diagnosed lipodystrophy [37]. This divergence could reflect difficulties in assessing lipodystrophy using single DXA scans, or suPAR not being associated with lipodystrophy in this more heterogeneous patient cohort. Please note that the sentences following, “Bonnet et al. [28] proposed reference values to define lipodystrophy by DXA scan. Only four of 283 patients in this study had lipodystrophy when applying this definition, indicating that it is not sensitive enough,” should be deleted.

The last sentence of the sixth paragraph of the Discussion section is incorrect. The sentence should read: However, we cannot exclude that the association of suPAR with low leg muscle mass is a physical activity-mediated effect.

The fourth sentence of the last paragraph of the Discussion section is incorrect. The sentence should read: However, we did not find any association with nadir CD4+ cell count, and suPAR was not significantly higher in individuals with low CD4+ cell count (<350 cells/μL).

Table 1 contains multiple errors. Please see the corrected Table 1 here.

**Table 1:** Baseline characteristics for HIV-infected patients not infected through intravenous drug use (IDU).

Demography	Median	Range (5%; 95% percentiles)	N total
Age (years)	44.3	29.5; 64.2	992
Sex (men)	74.9%		992
European descent	76.0%		992
**HIV-related Factors**			
HIV duration (years)	9.2	0.6; 21.8	992
Nadir CD4 (cells/μL)	183	9; 476	959
Nadir CD4<200 cells/μL	55.6%		959
Current cART	84.7%		990
Never cART	13.0%		990
Total treatment duration (years)	6.7	0.5; 10.8	861
CD4<350 cells/μL	21.2%		949
HIV RNA (copies/mL)	39	39; 33,300	947
HIV RNA≤40 copies mL	72.4%		947
**Lifestyle and Body Composition**			
Current daily smoking	35.1%		428
Waist circumference (cm)	91.5	72.0; 110.5	415
BMI (kg/m^2^)	23.9	19.1; 31.7	465
Metabolic syndrome	30.6%		366

**Abbreviations:** cART: Combination antiretroviral treatment; BMI: Body mass index

Table 2 Contains multiple errors. Please see the corrected Table 2 here.

**Table 2:** HIV- and non HIV-related factors influencing suPAR levels.

	Univariate				Multiple		
Variables	% Estimate (95% CI)		P	N	% Estimate (95% CI)	P	N
**Demography**							
Age ≥60 vs. <40 years	20.0 (10.6; 30.2)		<0.001	992	19.1 (9.4; 30.0)	<0.001	992
Age ≥60 vs. 40-50 years	9.3 (1.0; 18.3)				8.4 (0.1; 17.4 )		
Age ≥60 vs. 50-60 years	5.2 (-3.6; 14.8)				4.7 (-4.0; 14.3)		
Sex (men vs. women)	-1.8 (-6.9; 3.7)		0.52	992	-7.1 (-12.8; -1.1)	0.02	992
European descent	10.1 (4.4; 16.3)		<0.001	992	9.8 (3.1; 17.0)	0.004	992
**HIV-related**							
HIV duration (years)	0.5 (0.1; 0.8)		0.01	992	0.1 (-0.3; 0.5)	0.75	992
Nadir CD4+ (cells/μL)*	0.01 (-0.01; 0.02)		0.44	959	-0.01 (-0.02; 0.01)	0.62	958
No current cART*	9.0 (2.2; 16.2)		0.009	990	17.3 (8.0;27.4)	<0.001	958
Treatment duration (years)**	0.3 (-0.4; 1.0)		0.43	861	-1.4 ( -2.3; -0.4)	0.006	845
CD4<350 vs. 350≥cells/μL***	9.1 (2.8; 15.6)		0.004	949	5.7 (-0.9; 12.9)	0.09	912
VL, cART-treated patients (pr. 10-fold)***	14.7 (9.4; 20.3)		<0.001	795	15.4 (9.9; 21.1)	<0.001	780
VL, for untreated patients (pr. 10-fold)***	4.1 (-2.2; 10.9)		0.20	150	2.0 (-5.3; 9.9)	0.60	132
**Lifestyle and Body Composition**							
Daily vs. no daily smoking	26.4 (18.3; 35.1)		<0.001	428	26.7 (18.6; 35.4)	<0.001	428
Waist circumference (cm)#	0.2 (-0.04; 0.5)		0.09	415	0.3 (0.02; 0.6)	0.03	408
BMI <20 vs. 20-25 (kg/m^2^)##	2.9 (-6.5; 13.2)		0.01	483	-0.8 (-8.8; 11.4)	0.07	408
BMI <20 vs. 25-30 (kg/m^2^)##	11.9 (1.0; 23.9)				7.5 (-5.3; 22.0)		
BMI <20 vs. ≥30 (kg/m^2^)##	-5.8 (-17.6; 7.9)				-6.6 (-22.4; 12.4)		
Metabolic syndrome#	6.6 (-1.8; 15.8)	0.13		366	8.4 (0.2; 17.2)	0.04	338

All multiple analyses are adjusted for sex, age, and European descent.

*Multiple analyses are als° adjusted for time since HIV-diagnosis.

^**^Multiple analyses are also adjusted for time since HIV-diagnosis, current treatment and nadir CD4+ cell counts.

^***^Multiple analyses are also adjusted for time since HIV-diagnosis, nadir CD4+ cell counts, current treatment, CD4+<350 vs. ≥350 cells/μL, and log_10_(VL).

# Multiple analyses are also adjusted for daily smoking.

## Multiple analyses are also adjusted for daily smoking, waist circumference.

**Abbreviations:** cART: Combination antiretroviral treatment; VL: viral load; BMI: Body mass index; CI: Confidence interval.

Table 3 contains multiple errors. Please see the corrected Table 3 here.

**Table 3:** Subgroup analyses of body composition and suPAR levels in patients with DXA scan.

	Univariate			Multivariate		
Body Composition	% Estimate (95% CI)	P	N	% Estimate (95% CI)	P	N
Lean mass/h^2^ (kg/m^2^)	-1.8 (-3.4; -0.1)	0.04	283	-2.3 (-4.2; -0.4)	0.02	283
Lean mass_leg_/h^2^ (kg/m^2^)	-9.0 (-13.0; -4.9)	<0.001	283	-9.1 (-13.3; -4.8)	<0.001	283
Fat mass/h^2^ (kg/m^2^)	0.1 (-1.4; 1.6)	0.93	283	1.3 (-0.6; 3.3)	0.17	234
Total fat%	0.1 (-0.4; 0.5)	0.76	283	0.4 (-0.2; 1.1)	0.19	234
Limb fat%	0.1 (-0.3; 0.5)	0.70	283	0.4 (-0.2; 1.1)	0.20	234
Trunk fat %/leg fat %	-3.7 (-11.7; 5.0)	0.40	283	-4.2 (-20.7; 9.6)	0.37	234

Multiple analyses of lean mass measures are adjusted for sex, age and European descent. Multiple analyses of fat mass measures are adjusted for sex, age, European descent, and daily smoking.

**Abbreviations:** CI: Confidence interval; h = height.

Figure 2 and the associated legend are incorrect. The authors have provided a corrected version here.
